# From an Hsp90 - binding protein to a peptide drug

**DOI:** 10.1093/femsml/uqac023

**Published:** 2022-12-13

**Authors:** Aparna Viswanathan Ammanath, Anders Jarneborn, Minh-Thu Nguyen, Laura Wessling, Paula Tribelli, Mulugeta Nega, Christian Beck, Arif Luqman, Khaled A Selim, Hubert Kalbacher, Boris Macek, Sandra Beer Hammer, Tao Jin, Friedrich Götz

**Affiliations:** Microbial Genetics, Interfaculty Institute of Microbiology and Infection Medicine Tübingen (IMIT), University of Tübingen, Tübingen 72076, Germany; Department of Rheumatology and Inflammation Research, Institute of Medicine, Sahlgrenska Academy, University of Gothenburg, Gothenburg 41346, Sweden; Section of Medical and Geographical Infectiology, Institute of Medical Microbiology, University Hospital of Münster, Münster 48149, Germany; Department of Pharmacology, Experimental Therapy and Toxicology, Institute of Experimental and Clinical Pharmacology and Pharmacogenomik and ICePhA, University of Tuebingen, Tuebingen 72074, Germany; Departamento de Química Biológica, FCEyN-UBA, IQUIBICEN, Ciudad Autónoma de Buenos Aires, CP1428EGA, Argentina; Microbial Genetics, Interfaculty Institute of Microbiology and Infection Medicine Tübingen (IMIT), University of Tübingen, Tübingen 72076, Germany; Infection Biology, Interfaculty Institute for Microbiology and Infection Medicine Tübingen, University of Tübingen, Tübingen 72076, Germany; Biology Department, Institut Teknologi Sepuluh Nopember, Surabaya 60111, Indonesia; Organismic Interactions Department, Interfaculty Institute for Microbiology and Infection Medicine, University of Tübingen, Tübingen 72076, Germany; Excellence Cluster 2124 'Controlling Microbes to Fight Infections' (CMFI), University of Tübingen, Tübingen 72076, Germany; Interfacultary Institute for Biochemistry, University of Tübingen, Tübingen 72076, Germany; Proteome Center Tübingen, University of Tübingen, Tübingen 72076, Germany; Department of Pharmacology, Experimental Therapy and Toxicology, Institute of Experimental and Clinical Pharmacology and Pharmacogenomik and ICePhA, University of Tuebingen, Tuebingen 72074, Germany; Department of Rheumatology and Inflammation Research, Institute of Medicine, Sahlgrenska Academy, University of Gothenburg, Gothenburg 41346, Sweden; Microbial Genetics, Interfaculty Institute of Microbiology and Infection Medicine Tübingen (IMIT), University of Tübingen, Tübingen 72076, Germany; Excellence Cluster 2124 'Controlling Microbes to Fight Infections' (CMFI), University of Tübingen, Tübingen 72076, Germany

**Keywords:** Hsp90-interaction, infection, Lpl-derived peptides, larvae/mouse Infection model, *Staphylococcus aureus*

## Abstract

The Lpl proteins represent a class of lipoproteins that was first described in the opportunistic bacterial pathogen *Staphylococcus aureus*, where they contribute to pathogenicity by enhancing F-actin levels of host epithelial cells and thereby increasing *S. aureus* internalization. The model Lpl protein, Lpl1 was shown to interact with the human heat shock proteins Hsp90α and Hsp90ß, suggesting that this interaction may trigger all observed activities. Here we synthesized Lpl1-derived peptides of different lengths and identified two overlapping peptides, namely, L13 and L15, which interacted with Hsp90α. Unlike Lpl1, the two peptides not only decreased F-actin levels and *S. aureus* internalization in epithelial cells but they also decreased phagocytosis by human CD14^+^ monocytes. The well-known Hsp90 inhibitor, geldanamycin, showed a similar effect. The peptides not only interacted directly with Hsp90α, but also with the mother protein Lpl1. While L15 and L13 significantly decreased lethality of *S. aureus* bacteremia in an insect model, geldanamycin did not. In a mouse bacteremia model L15 was found to significantly decreased weight loss and lethality. Although the molecular bases of the L15 effect is still elusive, *in vitro* data indicate that simultaneous treatment of host immune cells with L15 or L13 and *S. aureus* significantly increase IL-6 production. L15 and L13 represent not antibiotics but they cause a significant reduction in virulence of multidrug-resistant *S. aureus* strains in *in vivo* models. In this capacity, they can be an important drug alone or additive with other agents.

## Teaser

L15 and L13 binds not only to Hsp90 of host cells but also to Lpl lipoproteins of *Staphylococcus aureus* thereby blocking the Lpl-Hsp90 interaction. This leads to a decrease of Lpl-Hsp90 mediated *S. aureus* invasion by keratinocytes, decrease of F-actin formation and decrease of *S. aureus* virulence. The decrease in virulence is indicated by an approximately 40% decrease in killing activity of *S. aureus* in both insect and mouse systemic infection model.

## Introduction

The opportunistic human pathogen *Staphylococcus aureus* can cause severe community acquired and nosocomial infections. Different proteins on the bacterial surface support its adherence to and/or internalization into host cells by either directly binding to a defined host cell receptor or by interacting with a matrix protein such as fibronectin, which in turn binds to the α5β1 integrin on the host cell surface (Sinha et al. 1999, Hirschhausen et al. 2010).

Among the cell envelope-bound proteins, the bacterial lipoproteins (Lpp) represent a distinct class of proteins. They are anchored to the outer leaflet of the cytoplasmic membrane by an N-terminal lipid moiety (Nguyen and Götz 2016); in Gram-negative bacteria they can in addition be anchored to the inner leaflet of the outer membrane (Buddelmeijer 2015). Many lipoproteins are part of an ABC transport system that takes up nutrients or have an essential role in respiration or protein folding (Shahmirzadi et al. 2016).

*Staphylococcus aureus* and few other species have so-called Lpl lipoproteins which are typically encoded in a cluster of paralogous genes on the pathogenicity island νSaα. Depending on the strain, 3 to 9 *lpl* genes may be encoded in this island (Diep et al. 2006, Nguyen et al.^2015^, Nguyen et al. 2016, Nguyen et al. 2016). As classical lipoproteins Lpl proteins have a typical lipo-box signal peptide and contribute to activation of the innate immune system *via* the toll like receptor 2 (TLR2) pathway. However, the function of their protein moiety has been long unknown. Only in the last few years it emerged that the *lpl* gene cluster is involved in the interaction of *S. aureus* with host cells and in the virulence of *S. aureus*.

When the entire *lpl* gene cluster was deleted, both, adherence to host cells and internalization of *S. aureus* by epithelial cells and keratinocytes were impaired (Nguyen et al. 2015, 2018). Additionally, in a murine kidney abscess model, mice challenged with the *S. aureus lpl* mutant displayed a reduced bacterial burden in the kidneys, indicating that the *lpl* cluster contributes to virulence. Further interaction studies using purified Lpl1, that is, the first encoded Lpl protein in the cluster, revealed that Lpl1 acts as a cyclomodulin by delaying G2/M phase transition in HeLa cells (Nguyen et al. 2016). However, unlike other staphylococcal cyclomodulins, such as phenol-soluble modulins (PSM) (Deplanche et al. 2015), Lpl1 shows no cytotoxicity even at high concentrations. Since internalization and propagation of *S. aureus* within the host cells takes place in the G2 phase (Alekseeva et al. 2013), an extension of the latter by the Lpl proteins could explain their contribution to host cell invasion on the molecular level (Nguyen et al. 2018).

A breakthrough in understanding the function of this subclass of lipoproteins was achieved when pull-down experiments revealed that the Lpl1 binds preferentially to the isoforms of the human heat shock proteins Hsp90α and Hsp90ß (Tribelli et al. 2020). Antibodies against Hsp90 decreased *S. aureus* invasion in primary human keratinocytes as well as in immortalized ones (HaCaT cells). Additionally, inhibition of the ATPase function of Hsp90 or silencing of Hsp90α expression by siRNA also decreased *S. aureus* invasion in HaCaT cells. While Hsp90ß is constitutively expressed, the Hsp90α isoform is heat inducible and appears to play a major role in the interaction with Lpl1. Pre-incubation of HaCaT cells at 39°C increased both Hsp90α expression and *S. aureus* invasion. Lpl1-Hsp90 interaction induces F-actin formation thus triggering an endocytosis-like internalization (Tribelli et al. 2020). Such a mechanism based on Lpl-Hsp90 interaction represents a new strategy of host cell invasion.

Geldanamycin, for example, inhibits the essential ATPase activity of Hsp90, resulting in the inactivation, destabilization and degradation of Hsp90 client proteins. Since these processes play an important role in the regulation of cell cycle, cell growth, cell survival, apoptosis and oncogenesis, geldanamycin inhibits the proliferation of cancer cells and shows anti-cancer activity in animal studies (Miyata 2005). Nonetheless, its pharmaceutical application is limited by its high cytotoxicity (Blagosklonny 2002, Miyata 2005).

The aim of this study was to identify domain sequences of the Lpl1 protein that interact with Hsp90α. For this purpose, we synthesized peptides covering the entire Lpl1 protein. We identified two peptides, L13 and L15, which interacted with Hsp90α and Lpl1, inhibited the internalization of *S. aureus* by host cells, and provided partial protection against *S. aureus* infection in insect and mouse infection models.

## Materials and methods

### Bacterial strains and cell lines

N/TERT-1 cells were a kind gift from Dr J Rheinwald, Harvard Medical School, Boston, USA (Dickson et al. 2000). N/TERT-1 cells were cultured in 24 well plates with keratinocyte serum-free medium (K-SFM) (Gibco, Invitrogen Corp.), supplemented with bovine pituitary extract (BPE) (25 μg per ml), EGF (0.2 ng per ml), and CaCl_2_ (0.4 mM). HaCaT cells were maintained in Dulbecco's Modified Eagle Medium (DMEM) (Thermo Fisher, Waltham, MA, USA) supplemented with 10% fetal bovine serum (FBS) (BiochromAG, Berlin, Germany) and 1% penicillin-streptomycin (Thermo Fisher, Waltham, MA, USA). MONO-MAC-6 (MM6) cell lines were maintained in Gibco Roswell Park Memorial Institute 1640 Medium (RPMI 1640) (Thermo Fisher, Waltham, MA, USA) supplemented with 10% FBS, 2 mM L-glutamine, non-essential amino acids, 1 mM sodium pyruvate and 10 µg/ml human insulin. *S. aureus* Newman and USA300 were cultured aerobically in Tryptic Soy Broth (TSB) at 37°C. The bacterial strains were stored at −70°C. Upon aerobic culture, bacterial cells were washed with sterile phosphate-buffered saline (PBS), and adjusted to the desired concentration.

### Peptide synthesis

The amino acid sequences of peptides used in this study are given in Table S1. The peptides were synthesised by Apeptide (Shanghai, China) with a purity of >95%, dissolved in water at 1 mg/ml and stored at −20°C. 20 µM of L15, 30 µM of L13 and 5 µM of geldanamycin were used in *in vitro* studies unless described otherwise.

### Invasion studies in HaCaT and N/TERT-1 cells

The invasion protocol was adapted from our previous papers (Nguyen et al. 2015, Tribelli et al. 2020). About 5 × 10^5^ Keratinocytes were seeded in a 24 well plate (Greiner, Frickenhausen, Germany) to attain a monolayer of ∼10^6^ cells/well. *Staphylococcusaureus* was grown overnight at 37°C, centrifuged and suspended in invasion medium (DMEM with 10% FBS for HaCaT and K-SFM for NTERT-1 cells). The adherent keratinocytes were washed with PBS and supplemented with invasion medium followed by treatment with 20 µM of L15, 30 µM of L13, or 5 µM geldanamycin (Sigma-Aldrich, Germany) or monoclonal antibodies specific against Hsp90α (α‐Hsp90α, Abcam) for 1 h. After 1 h, 100 µl of bacterial suspension was added to each well to attain a MOI (multiplicity of infection) of 30 and incubated with cells for 1.5 h. Subsequently, the extracellular bacteria were killed with treatment of 2.5 μg/ml lysostaphin (Sigma-Aldrich, Germany) for 1.5 h. The cells were then washed, lysed, mechanically detached by scraping, diluted, and enumerated on TSA (Tryptic Soy Agar) plates to quantify the intracellular bacteria.

### CD14^+^ monocyte isolation

Peripheral Blood Mononuclear cells (PBMCs) were isolated by density gradient centrifugation following the previous study (Nguyen et al. 2022). From the pool of PBMCs, monocytes were isolated by positive selection with anti-human CD14 microbeads (Miltenyi Biotech, Bergisch-Gladbach, Germany) following a previously reported protocol (Nguyen et al. 2022). The purity was analysed by flow cytometry on a BD Accuri C6 (BD Biosciences, Heidelberg, Germany) with anti-human CD14-FITC, CD45-PE and propidium iodide (BD Biosciences, Heidelberg, Germany) and ranged from 85% to 98%.

### Phagocytosis assay

For the phagocytosis, 10^6^ CD14^+^ monocytes were seeded in 1 ml of medium (RPMI supplemented with L-Glutamine and 10% FCS) in a 12 well plate (Greiner, Germany). The cells were incubated with 20 µM of L15, 30 µM of L13, or 5 µM of Geldanamycin for 60 min at 37°C and 5% CO2 before addition of bacteria. For the phagocytosis assay, bacterial cells were resuspended in 100 µl RPMI medium at MOI of 30 and incubated with monocytes for 90 min. The cells were washed once with PBS and 0.5 ml of medium supplemented with 2.5 µg/ml of Lysostaphin was added for 90 min to remove the extracellular bacteria. Then, monocytes were washed twice with PBS and resuspended in 0.5 ml of milliQ dH2O. The cells were scraped, and the lysed solution was transferred into a new 1.5 ml Eppendorf tube for a 5 min sonication (frequency 80, power 100) at room temperature to prevent the bacterial cell clumping by using ultrasonic water bath Elmasonic P (Elma Schmidbauer Gmb, Singen, Germany). About 10 µl fractions of undiluted, 10^−1^, 10^−2^, and 10^−3^ dilutions were inoculated on tryptic soy agar (TSA) plates and incubated overnight at 37°C. The numbers of internalized bacteria were determined based on the manual counting of bacterial colony forming units (cfu) recovered on the agar plates.

### Bacterial growth kinetics

*Staphylococcus aureus* precultured in TSB overnight were inoculated to OD∼0.01 into a 48 well plate and L15 and L13 were added to study the effect of peptides on bacterial growth using Varioskan LUX Multimode Microplate Reader. With this instrument, a kinetic measurement of OD_578 nm_ was obtained every 1 h for a total of 24 h, at 37°C with continuous shaking.

### Peptide—Hsp90α/Lpl1 interaction studies with immunoblotting

Two µg of each peptide was blotted (dot blot) directly on PDV nitrocellulose membrane and blocked with ROTI®Block (Carl Roth, Germany). After washing, the membrane was then incubated with 20 μg of recombinant Hsp90α (Abcam)/Lpl1-his protein at 4°C overnight. In case of interaction studies with Hsp90α, α-Hsp90α (produced in mouse, Abcam) was used as the primary antibody and for interaction studies with Lpl1-his, Anti-6X His tag antibody (also produced in mouse, Sigma-Aldrich, Germany) was used. Alkaline phosphatase conjugated goat-α-mouse IgG (Sigma-Aldrich, Germany) served as the secondary antibody. Detection was done using BCIP®/NBT solution (Sigma, Germany). Lpl1-his was isolated and purified as described in our previous paper (Tribelli et al. 2020).

### F-actin measurement

About 5 × 10^4^ cells were seeded in black flat bottom 96 well cell culture microplate (Greiner, Germany) and incubated overnight. The cells were then incubated with peptides or geldanamycin for 1.5 h. After incubation, the cells were washed, permeabilised with 0.1% (v/v) Triton X-100 and stained with ActinGreen™ 488 ReadyProbes® (Thermo Fischer, Germany) for 30 min. After the staining, the cells were washed and measure for its fluorescence at Excitation/Emission: 495/518 nm (Luqman et al. 2020).

### Cytotoxicity studies

MTT (3-(4,5-dimethylthiazol-2-yl)-2,5-diphenyl tetrazolium bromide) assay was employed to analyze the cytotoxicity of peptides and geldanamycin to the cells. 5 × 10^5^ cells were seeded in a 96 flat bottom well plate and incubated for 2.5 or 24 h at 37°C with 5% CO_2_. The cells were then treated with increasing concentration (0 to 200 µM) of peptides or geldanamycin for 24 h. After the incubation, 10 μl of the MTT labelling reagent is added to each well to attain a final concentration 0.5 mg/ml, followed by 4 h incubation. Metabolically active cells convert the yellow tetrazolium salt to purple formazan crystals, which were solubilized using the solubilization solution (DMSO) and measured for absorbance at λ_max_/λ_ref–_570/690 nm (Saising et al. 2018).

### Galleria mellonella larvae studies

*Galleria mellonella* larvae, purchased from Feeders & more GmbH, Germany, were sorted based on weight and used within one week. Ten larvae per group with weight average of 500 mg/larvae were injected with bacteria and/or peptides on its last proleg using a BD insulin syringe. The dosage used for the experiment were 60 mg/kg for peptides, 5 mg/kg of geldanamycin and 20 mg/kg for vancomycin. Each larva was injected with 10 µl of L15 (last left proleg) 1 h before administration of 10^6^ cfu *S. aureus* (last right proleg). The larvae were maintained at 37°C and observed for mortality every day over the course of 5 days.

### Hemolysin activity

About 6 mm Whatman empty Antibiotic Assay Discs were impregnated with L15 or L13 and allowed to dry. Once dried, the discs were placed on sheep blood agar plates, inoculated with *S. aureus* and incubated at 37°C overnight. The halo obtained was measured and compared.

### Mouse studies

About 8- to 10-week-old female NMRI mice were purchased from Envigo (Venray, the Netherlands) and stored under standard temperature, light, and nutrition conditions in the animal facility of the Department of Rheumatology and Inflammation Research, University of Gothenburg. Due to its poor solubility in PBS, L13 was not used in mouse experiments. To study the effect of L15 on *S. aureus* bacteremia two separate experiments were performed. The mice were treated intraperitoneally with L15 (10 mg/kg) in 200 µl of PBS every 12 h, starting 2 h before inoculation with bacteria and continuing until the animals were sacrificed. Mice were challenged with septic dose of 2 × 10^6^ CFU/mouse of *S aureus* Newman. To induce the *S. aureus* systemic infection in NMRI mice, 0.2 ml of *S. aureus* Newman suspension was inoculated intravenously into the tail vein of the mice. During the course of the experiments, the mice were regularly weighed until the mice were sacrificed. After sacrificing the mice at day 7, kidneys from mice were aseptically removed, homogenised, serially diluted with PBS and enumerated to quantify the bacterial CFUs.

### Immune stimulation

Human peripheral blood mononuclear cells (PBMCs) from buffy coat blood samples of healthy donors were stimulated with the indicated doses of L15 and L13 for 20 h. For the subsequent bead-based immunoassay, the cell culture supernatants of a total of four buffy coats per immunoassay were used. Each of the stimulations was performed in biological duplicates. The secreted cytokines were then identified and quantified using the immunofluorescent bead-based immunoassay LEGENDplex® Human Macrophage/Microglia Panel (13-plex) and the corresponding Data Analysis Software from BioLegend. Muramyl Dipeptide (MDP) which activates the NOD-2 receptors was used as the positive control.

### Bioinformatics analysis

The 3D structure of Lpl1 was predicted using the protein structure prediction server Robetta using RoseTTAFold modelling method (Baek et al. 2021). The obtained .pdb structure was then visualized in PyMOL (Schrödinger). The homologs of *S. aureus* USA300 Lpl1 in different bacteria were identified using the Protein BLAST tool on the NCBI server. The comparison of different lipoprotein homologs from different bacteria was done in Snapgene using Clustal Omega algorithm.

### Statistical analysis

GraphPad Prism was employed for statistical analysis. Student's t tests or one-way analysis of variance (ANOVA) were used to check the statistical significance of different results. *P-*value > 0.05 was considered as not significant (ns). In figures, significant differences are represented as follows: * *P* < 0.05; ** *P* < 0.01; *** *P* < 0.001; and ^****^P < 0.0001.

### Ethical statement

The use of human peripheral blood mononuclear cells (PBMCs) from buffy coats was approved by the Ethics Committee of the Medical Association of Westphalia-Lippe and the University of Münster (Approval number 2021–063-f-S) and the Ethics Committee of the Medical Faculty of the University of Tübingen and the Medical Clinic Tübingen (approval number 084/2021BO2). Buffy coats were obtained from the German Red Cross Blood transfusion west (Hagen, Germany) and the Transfusion Blood Bank of the Medical Clinic Tübingen. The Ethics Committee of Animal Research of Gothenburg approved all experiments conducted on mice. The mouse experiments were performed in accordance with the Swedish Board of Agriculture's regulations and recommendations on animal experiments.

## Results

### Lpl1-derived peptides L15 and L13 decrease *S. aureus* USA300 internalisation by keratinocytes in a Hsp90 mediated way

**Figure 1.**
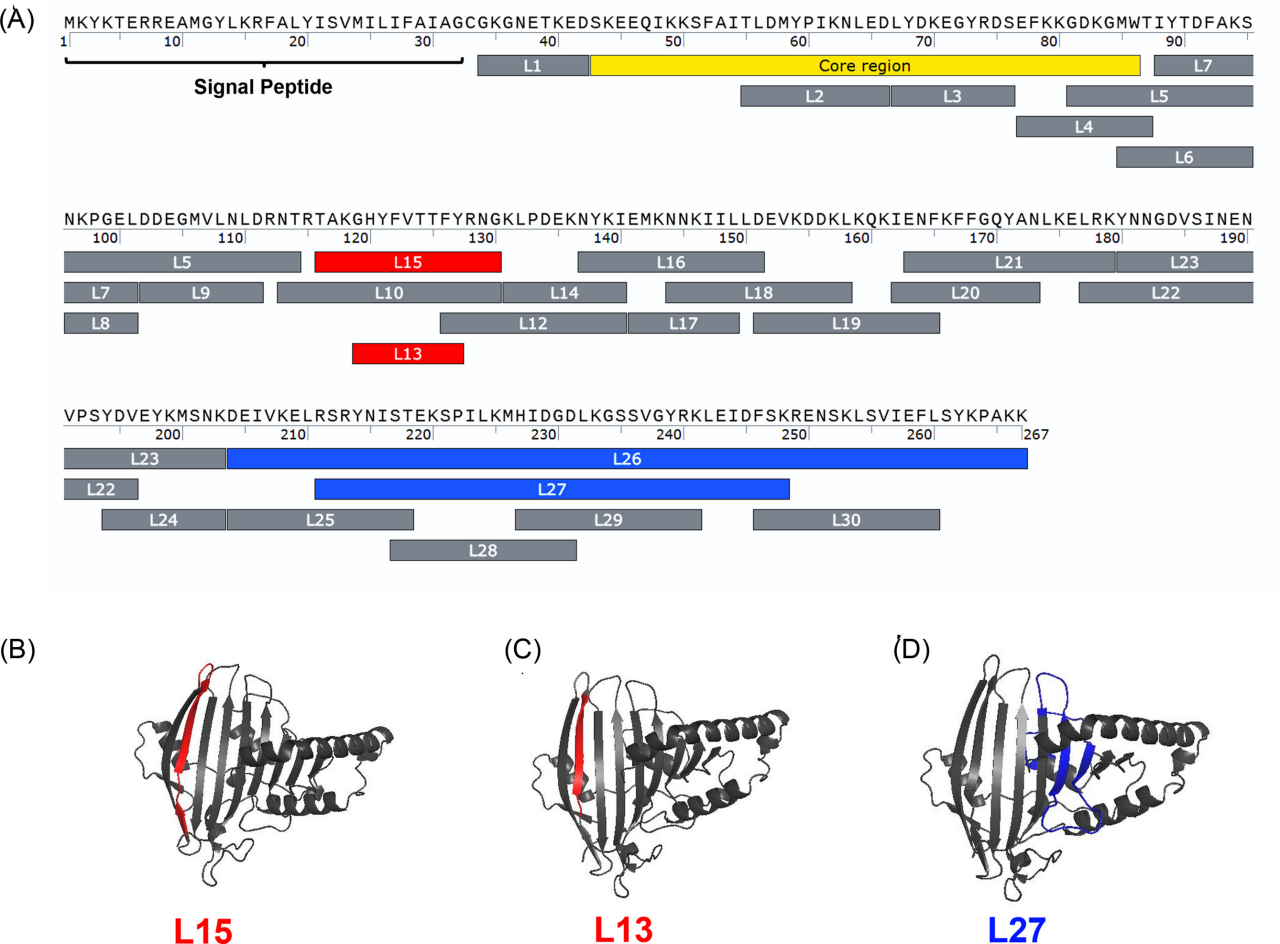
Schematic overview of the smaller peptide fragments of Lpl1 and their invasion activity in host cells. (**A**) Representation of the peptide fragments used in the study. L15 and L13, labelled in red, reduced *S. aureus* internalization in HaCaT cells. L26 an L27, labelled in blue, increased *S. aureus* internalization. Most of the other synthesized peptides showed no effect in *S. aureus* internalization in HaCaT cells (labelled in grey). Localisation of (**B**) L15 (highlighted in red), (**C**) L13 (highlighted in red) and (**D**) L27 (highlighted in blue) in the Lpl1-protein. The 3D structure of Lpl1 was predicted using the protein structure prediction server Robetta using RoseTTAFold modelling method and visualized in PyMOL.

In our previous work, we demonstrated that the *lpl* cluster of *S. aureus* USA300 triggers invasion of *S. aureus* into HaCaT cells. In the USA300Δ*lpl* mutant, the rate of invasion was 2.5-fold lower compared with wild type (Nguyen et al. 2016). We identified two Lpl1-derived peptide sequences (L26 and L27) that boosted *S. aureus* internalization to the same extent as the whole Lpl1 protein (blue-labelled peptide regions in Fig. 1A).

**Figure 2.**
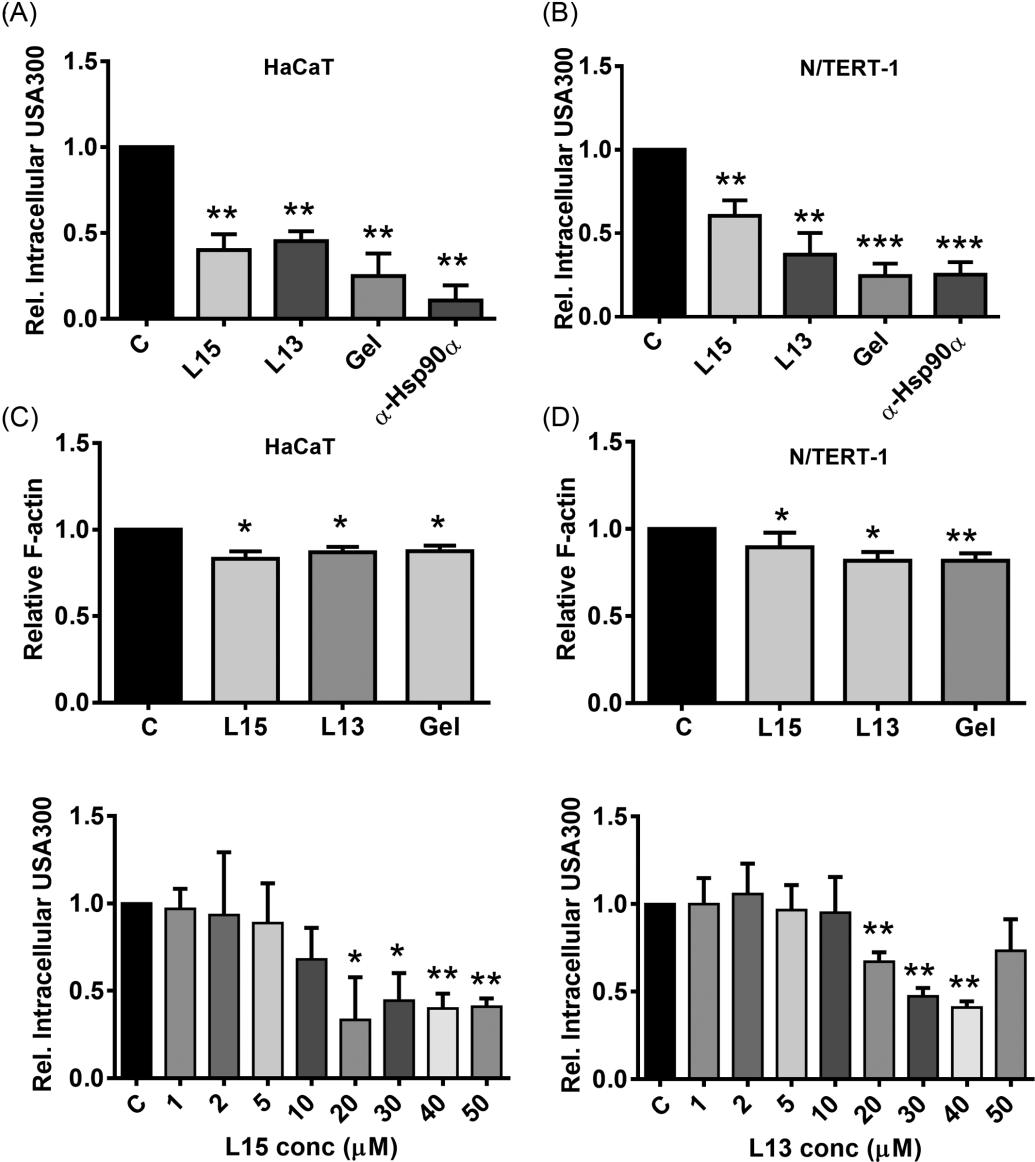
Bioactivity of L15 and L13 with respect to invasion and F-actin levels. One-hour-half long pretreatment (1.5 h) with L15 and L13 inhibits the invasion of *S. aureus* USA300 into (**A**) HaCaT and (**B**) N/TERT-1 cell lines. Pretreatment of cells with geldanamycin (Gel), a known Hsp90 inhibitor, or with α-Hsp90α (Hsp90α) antibody also reduced USA300 invasion into the keratinocytes. L15, L13 and geldanamycin reduced F-actin levels in (**C**) HaCaT and (**D**) N/TERT-1 cells. The cells were treated with 20 µM of L15, 30 µM of L13 or with 5 µM of geldanamycin for these analyses. C indicates control. Error bars show standard deviation of the mean of 3 biological replicates. *P-*values were obtained by student's T-test: * *P* < 0.05; ** *P* < 0.01; *** *P* < 0.001; and ^****^P < 0.0001.

Here, we further dissected the Lpl1-protein into smaller peptide fragments and examined these for their invasive activity by host cells. All Lpl1-derived peptides tested are marked in Fig. 1A. Most of the synthesized peptides showed no effect (grey-labeled peptide regions in Fig. 1A). However, we found two small peptides that produced the opposite effect, namely, they decreased *S. aureus* internalization by host cells (red-labeled peptide regions in Fig. 1A). We named these two peptides L13 and L15. L13 is part of L15 and they are 9 and 15 amino acids long, respectively; (Fig. 1A). Both peptides are part of a beta-sheet region of the Lpl1-protein (Fig. 1B). Preincubation of HaCaT cells with L13 or L15 caused a 50–60% decrease in uptake of *S. aureus* USA300 compared to untreated control (Fig. 2A). As an additional control we included the well-known Hsp90 inhibitor geldanamycin (5 μM), a benzoquinoid from *Streptomyces hygroscopicus* that is known to inhibit the ATPase activity of Hsp90. As expected, geldanamycin reduced the internalization of *S. aureus* USA300 by HaCaT cells by approximately 75% (Fig. 2A). As a positive control, we also tested the peptides L26 and L27, which increased invasion by approximately 1.5-fold (Table 1). All other peptides had no effect on the internalization of *S. aureus* into HaCaT cells. (Please note, L26 and L27 were called P10 and P11, respectively in our previous paper (Tribelli et al. 2020)).

**Table 1.** Effect of tested Lpl1-derived peptides on their invasion potential, F-actin levels in HaCaT cells and binding to Hsp90α. In all the studies, 35 µg/ml of peptides were tested for its effect on HaCaT cells.

Name	Sequence	No of AA	Molarity (µM)	Relative Invasion in HaCaT	Interaction with Hsp90α	Relative F-actin level in HaCaT
L1	^34^ GKGNETKED	9	35.82	1.02 ± 0.34	No	0.95 ± 0.49
L2	^55^ TLDMYPIKNLED	12	24.14	1.12 ± 0.40	No	0.99 ± 0.13
L3	^67^ LYDKEGYRDS	10	28	1.00 ± 0.11	No	1.03 ± 0.1
L4	^77^ EFKKGDKGMWT	11	26.32	0.93 ± 0.12	No	0.94 ± 0.08
L5	^81^GDKGMWTIYTDFAKSNKPGELDDEGMVLNLDRNTR	34	8.77	1.59 ± 0.76	No	0.97 ± 0.08
L6	^85^ MWTIYTDFAKS	11	25.74	0.98 ± 0.68	No	0.91 ± 0.1
L7	^88^ IYTDFAKSNKPGEL	14	22.15	1.10 ± 0.18	No	1.03 ± 0.08
L8	^96^ NKPGEL	6	53.03	1.28 ± 0.34	No	0.96 ± 0.05
L9	^102^ DDEGMVLNLD	10	31.25	1.0 ± 0.44	No	1.06 ± 0.07
L10	^113^ NTRTAKGHYFVTTFYRNG	18	16.43	1.10 ± 0.17	Yes	0.99 ± 0.08
L13	^119^ GHYFVTTFY	9	30.97	0.45 ± 0.08	Yes	0.87 ± 0.03
L15	^116^ TAKGHYFVTTFYRNG	15	19.88	0.4 ± 0.09	Yes	0.83 ± 0.04
L12	^126^ FYRNGKLPDEKNYKI	15	18.52	0.94 ± 0.11	No	0.98 ± 0.06
L14	^131^ KLPDEKNYKI	10	28	0.78 ± 0.26	No	0.98 ± 0.08
L16	^137^ NYKIEMKNNKIILLD	15	18.91	0.92 ± 0.37	Yes	1.08 ± 0.23
L17	^141^ EMKNNKIIL	9	31.82	1.05 ± 0.26	No	1.09 ± 0.20
L18	^144^ NNKIILLDEVKDDKL	15	19.77	1.05 ± 0.36	No	1.09 ± 0.22
L19	^151^ DEVKDDKLKQKIENF	15	18.92	1.29 ± 0.37	No	0.97 ± 0.11
L20	^162^ IENFKFFGQYAN	12	23.65	1.15 ± 0.20	No	1.03 ± 0.11
L21	^163^ENFKFFGQYANLKELRK	17	16.43	1.20 ± 0.25	Yes	1.09 ± 0.13
L22	^177^LRKYNNGDVSINENVPSYDV	20	15.22	1.29 ± 0.13	No	0.97 ± 0.09
L23	^180^YNNGDVSINENVPSYDVEYKMSNK	24	12.59	1.09 ± 0.46	No	0.96 ± 0.05
L24	^194^YDVEYKMSNK	10	27.34	0.95 ± 0.12	No	0.94 ± 0.08
L25	^204^ DEIVKELRSRYNIST	15	19.23	1.0 ± 0.06	No	1.03 ± 0.16
L26	^204^DEIVKELRSRYNISTEKSPILKMHIDGDLKGSSVGYRKLEI DFSKRENSKLSVIEFLSYKPAKK	64	4.72	1.50 ± 0.17	Yes	1.25 ± 0.13
L27	^211^RSRYNISTEKSPILKMHIDGDLKGSSVGYRKLEIDFSKRENSK	38	7.03	1.55 ± 0.17	Yes	1.36 ± 0.12
L28	^217^STEKSPILKMHIDGD	15	20.96	1.0 ± 0.27	No	0.90 ± 0.04
L29	^227^HIDGDLKGSSVGYRK	15	21.47	1.21 ± 0.33	No	0.90 ± 0.05
L30	^246^FSKRENSKLSVIEFL	15	19.44	1.24 ± 0.32	No	0.96 ± 0.1
	Geldanamycin		5	0.25 ± 0.13	NA	0.88 ± 0.03
	Lpl1				Yes	
	BSA				No	

We also investigated whether L15 and L13 mediated internalization of *S. aureus* by the host cell is dependent on Hsp90α. For this purpose, we pretreated HaCaT cells with anti-Hsp90α (α-Hsp90α) antibody, which decreased *S. aureus* invasion by 89.3% (0.11 ± 0.09) (Fig. 2A). This suggested that the interaction of the peptide with Hsp90α plays a crucial role in host cell internalization.

Similar results were obtained with the N/TERT-1 keratinocyte cell line (Fig. 2B), which is often used as a substitute for primary keratinocyte cells because of the limited availability and high inter-donor variability of the latter. N/TERT-1 cells are immortalized and behave essentially like primary human keratinocytes in terms of host defense gene and protein expression and epidermal differentiation (Smits et al. 2017). It should be noted that the peptides/geldanamycin at the tested concentration and exposure time, didn't cause any toxicity either to HaCaT or N/TERT-1 cells (Fig. S1).

### L15 and L13 reduced F-actin levels in keratinocytes and directly interact with Hsp90α

In our previous work, we showed that the parent protein, Lpl1, increased F-actin levels in HaCaT cells (Tribelli et al. 2020), whereas geldanamycin (17-AAG) decreased F-actin levels in breast cancer cells (Taiyab and Rao Ch 2011). Here we investigated whether L15 and L13 influenced actin polymerization and found that both peptides, similarly to geldanamycin, caused a significant decrease in F-actin levels in both HaCaT and N/TERT-1 cell lines (Fig. 2C and D). In HaCaT cells L15 decreased the relative F-actin content to 0.83 ± 0.04, L13 to 0.87 ± 0.03, and geldanamycin to 0.88 ± 0.03. In N/TERT-1 cells we observed a similar decrease in F-actin levels for all three compounds, L13 (0.81 ± 0.03), L15 (0.9 ± 0.02) and geldanamycin (0.82 ± 0.03). As expected, L26 and L27, which induced increased host cell invasion, caused an increase in F-actin levels (Table 1).

A concentration dependent effect of the peptides L15 and L13 on *S.aureus* USA300 invasion into HACaT cells were carried out (from 0 to 50 µM). A significant reduction of intracellular USA300 were observed from a concentration of 20 µM on for both L15 and L13 (Fig. 2E and F). For L15, the relative invasion or intracellular bacterial count remained almost similar from concentration 20 to 50 µM (from 0.33 ± 0.24 to 0.40 ± 0.05) as compared to the untreated control (1.0 ± 0.0). In case of L13, a concentration dependent reduction in USA300 invasion into HaCaT were seen from 20 to 40 µM (0.67 ± 0.05 to 0.41 ± 0.03) as compared to the untreated group. At 50 µM L13, no significant change in the intracellular USA300 count was observed (0.73 ± 0.18).

**Figure 3.**
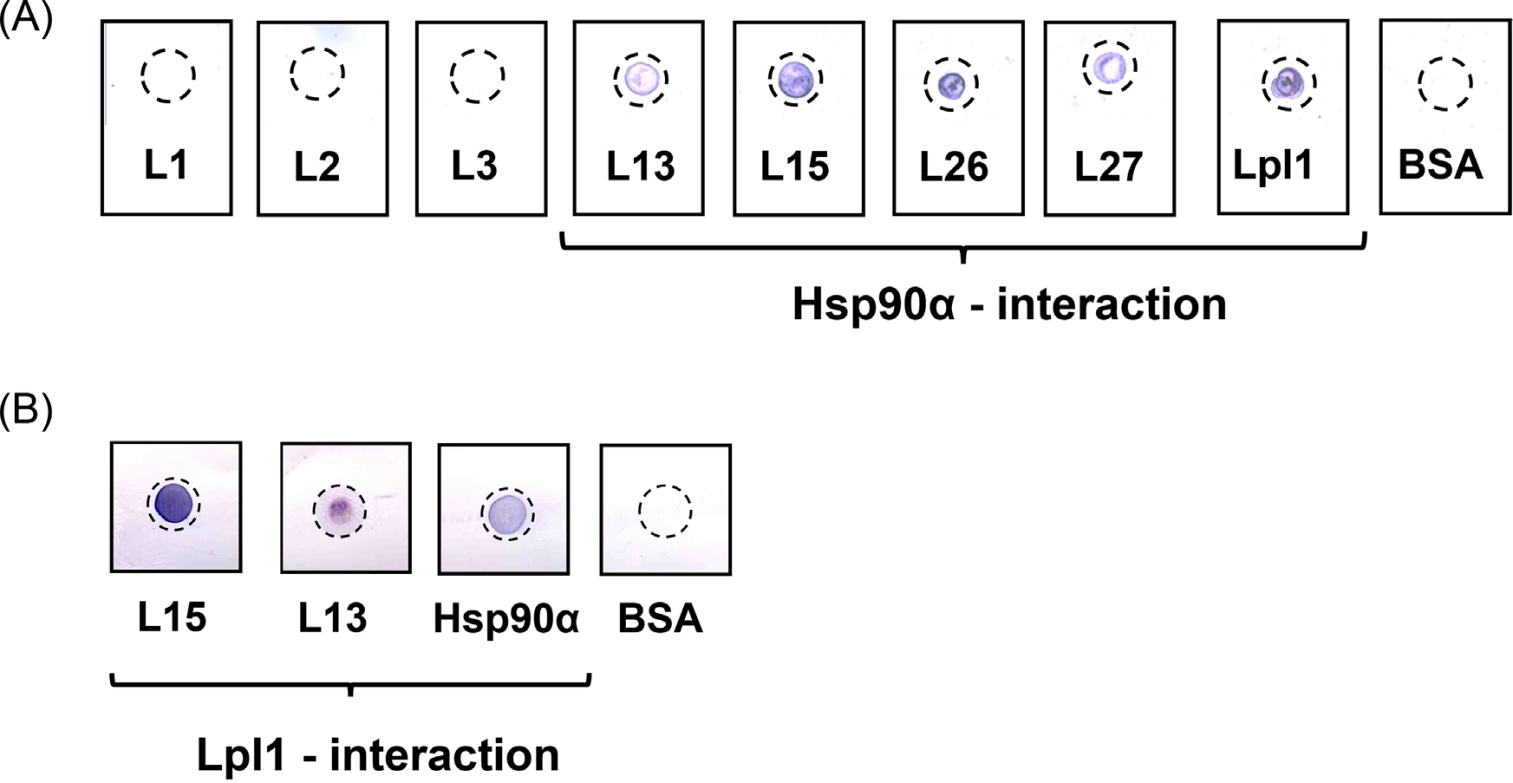
L15 and L13 interacts with Hsp90α and Lpl1 **(A)** Interaction of Lpl1 and Lpl1-derived peptides with Hsp90α by dot blot analysis. Here we showed that most of the Lpl1-derived peptides do not interact with Hsp90 as exemplified with L1 to L3. The C-terminal localized peptides L26 and L27 bind to Hsp90, but like the parent Lpl1 they boost internalization and F-actin levels. The L13 and L15 peptides also bind to Hsp90 but show an opposite effect. BSA was used as another negative control. **(B)** Dot blot image showing the direct interaction of L15, L13 with Lpl1 *via* immunoblotting. Hsp90α and BSA was used as the positive and negative control respectively.

In our previous work, we showed that Lpl1 interacts directly with Hsp90α (Tribelli et al. 2020). Here, we performed similar experiments with the synthetic peptides by blotting them directly onto PDV nitrocellulose membrane and testing for binding to Hsp90α *via* immunoblotting. The results are summarized in Table 1. Most of the Lpl1-derived peptides showed no binding to Hsp90α as exemplified by peptides L1 to L3. However, peptides that either promoted (L26 und L27) or inhibited (L13 and L15) invasion and F-actin levels also interacted with Hsp90α (Fig. 3A).

L15 and L13 were also observed to interact directly with their mother protein Lpl1, when tested with dot blot method. Here, 2 µg L15, L13 or Hsp90α was blotted directly on PDV nitrocellulose membrane and tested for their binding to Lpl1-His via immunoblotting. Hsp90α was used as positive and BSA as negative control (Fig. 3B).

### L15 and L13 are non-toxic to HaCaT and MM6 cells

**Figure 4.**
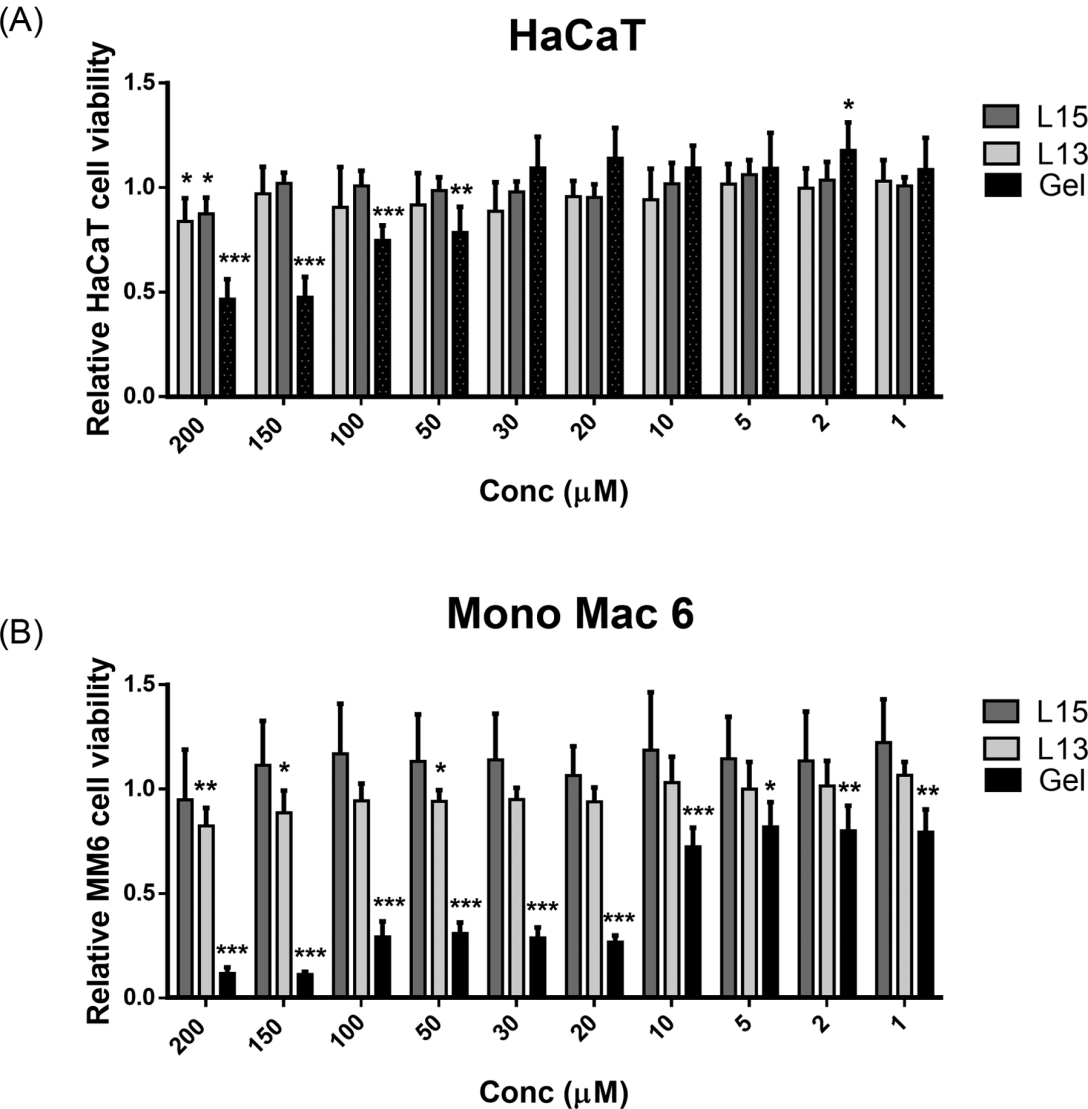
Unlike geldanamycin, L15 and L13 show little or no cytotoxicity. (**B**) HaCaT keratinocytes and (**C**) Mono Mac 6 (MM6) cells were incubated with increasing concentration (0–200 µM) of peptides or geldanamycin for 24 h and the viability of cells was determined using MTT cytotoxicity assay. Relative toxicity was calculated by normalising toxicity of peptides/geldanamycin treated cells to toxicity of untreated control cells. Error bars show standard deviation of the mean of three biological replicates. *P*-values were obtained by student's T-test: * *P* < 0.05; ** *P* < 0.01; *** *P* < 0.001.

Considering their potential as antimicrobials, we investigated whether L15, L13 and geldanamycin (as a control) are cytotoxic to human cells over a concentration range of 1–200 µM and for 24 h. Cytotoxicity was tested using the MTT assay in human keratinocytes (HaCaT cells) and human monocytic cells (MM6 cells). The percentage of viable cells after 24 h of treatment with **L15** ranged from 94.95 ± 24.03 (200 µM) to 122.27 ± 20.79 (1 µM) for MM6 cells and 87.39 ± 7.84 (200 µM) and 100.76 ± 4.20 (1 µM) for HaCaT cells. Cell viability upon **L13** exposure ranged between 82.43 ± 8.59% (200 µM) and 106.71 ± 6.40% (1 µM) in MM6 cells and 83.79 ± 11.13 (200 µM) and 103.05 ± 10.06 (1 µM) in HaCaT cells. Overall, it can be said that a slight reduction in viability was only observed at the highest concentration of 200 µM for both L13 and L15. In contrast, geldanamycin exhibited relatively high cytotoxicity also at lower concentrations (Fig. 4A and B). In HaCaT cells, viability decreased gradually from a concentration of 50 µM, and in MM6 cells already from 10 µM.

### L15 and L13 significantly decrease lethality of *S. aureus* bacteremia in insect model but they do not affect growth or hemolytic activity of *S. aureus*

**Figure 5.**
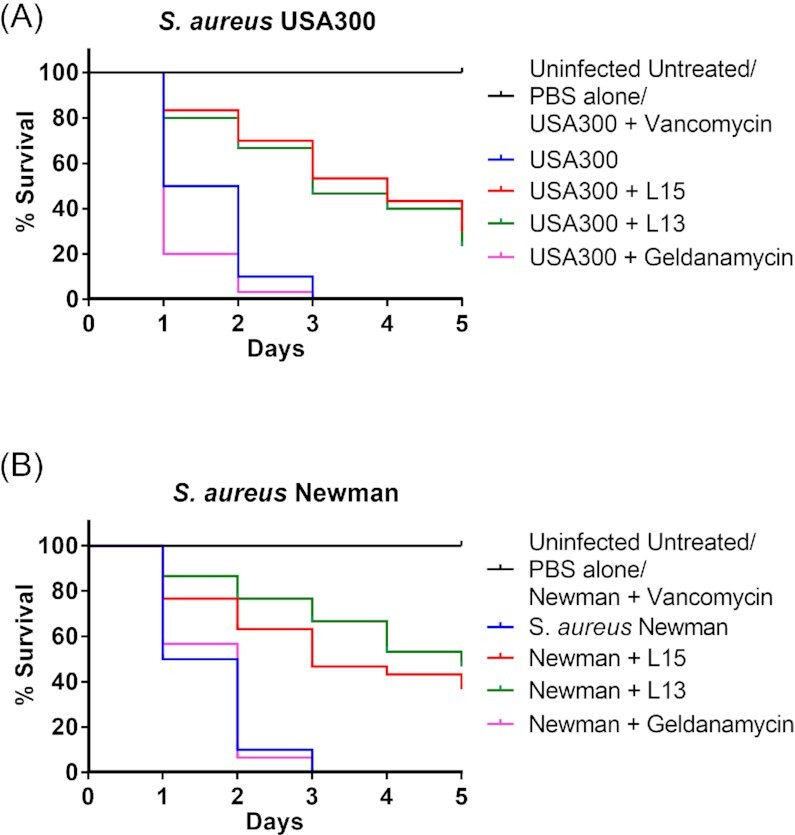
L15 and L13 rescue larvae from *S. aureus* infection. Ten *Galleria mellonella* larvae per group, with average weight of 500 mg/larvae, were injected with bacteria and/or peptides on their last proleg using a BD insulin syringe. Each larva was injected with 10 µl of either of the two peptides, or geldanamycin or vancomycin (last left proleg) 1 h before administration of 10^6^ colony forming units (cfu) *S. aureus* (last right proleg) (**A**) USA300 and (**B**) Newman. The dosages used for the experiment were 60 mg/kg for peptides, 5 mg/kg of geldanamycin and 20 mg/kg for vancomycin. The larvae were maintained at 37°C and observed for mortality every day over the course of 5 days. A total of three biological replicates are represented in the graph.

The larvae of *Galleria mellonella*, or *Greater Wax Moth*, are a recognized experimental model for studying the virulence of various pathogens and for evaluating the efficacy of antimicrobial compounds. The large size of the larvae ensures easy handling and direct injection of a drug into the larval hemocoel. Ten microliters of L15 (60 mg/kg), L13 (60 mg/kg) or geldanamycin (5 mg/kg) were injected into their hemocoel of *G. mellonella* 1 h prior to inoculation of *S. aureus* USA300 or *S. aureus* Newman, respectively. Larval survival was then followed for 5 days. Figure 5A shows the killing rate of larvae infected with *S. aureus* USA300 alone and treated with L15 (20 µM), L13 (30 µM), or geldanamycin (5 µM). All infected untreated larvae died by the end of the third day. However, when the larvae were pretreated with L15 or L13, 30% and 23% of the larvae still survived on day 5, respectively. When the larvae were infected with *S. aureus* Newman, the picture was similar. When pretreated with L15 or L13, 36% or 46% of the larvae, respectively, survived on day 5 (Fig. 5B). When pretreated with vancomycin (20 mg/kg), 100% of the larvae survived, whereas geldanamycin had no or rather a negative effect on survival. The tested doses of peptides, geldanamycin, and vancomycin were not toxic to larvae.

Since L15 and L13 could partially protect larvae from *S. aureus* infection, we then investigated whether they affected growth or the expression of virulence markers such as hemolysis. However, we did not detect any effect on growth (Fig. S2A), nor was hemolysis activity markedly affected (Fig. S2B).

### L15 significantly decrease lethality of *S. aureus* bacteremia in mice

The results seen in larvae infection model encouraged us to test the same protective effect in mice. Here we chose the better soluble peptide, L15. To test this, NMRI mice were intravenously infected with *S. aureus* Newman. The clinical outcomes were monitored during the course of a 7-day infection. L15 treated mice were compared with the control mice receiving PBS.

**Figure 6.**
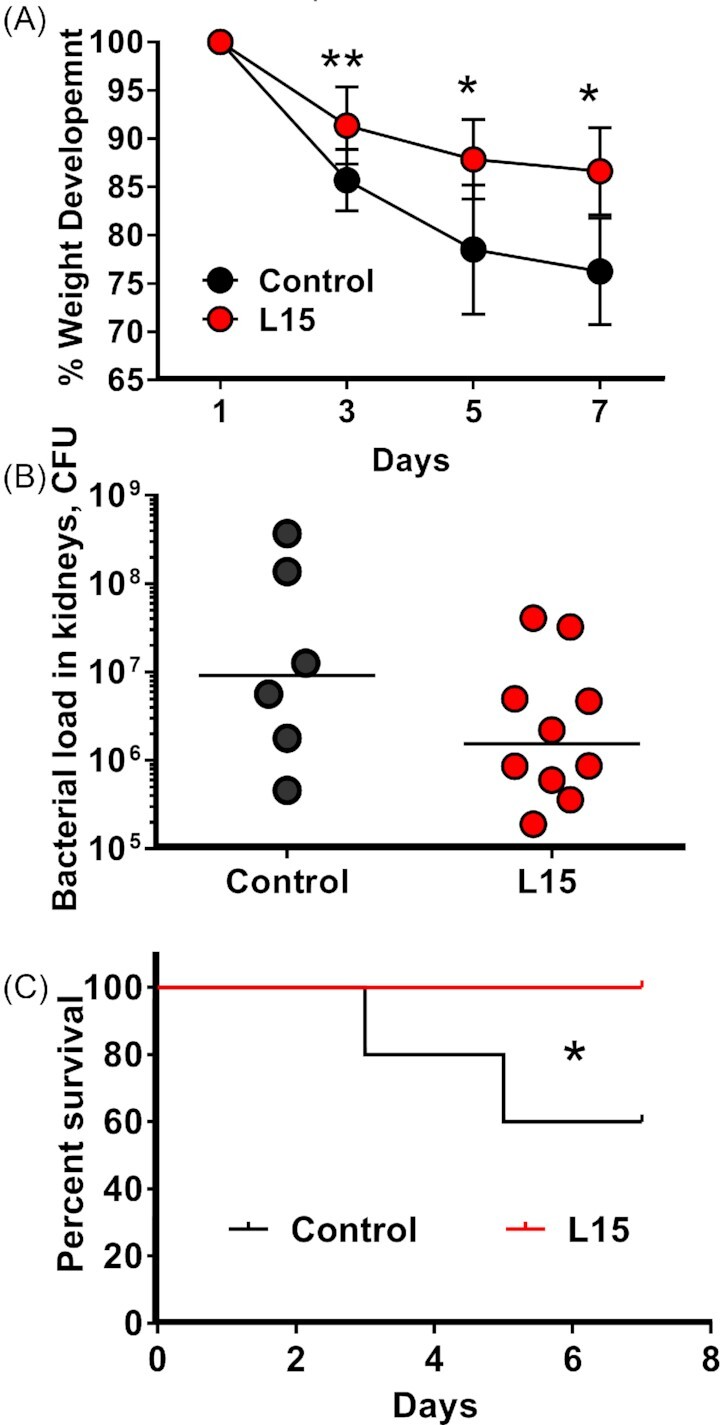
L15 treatment ameliorates systemic *S. aureus* infection in mice. NMRI mice were intraperitoneally treated with L15 (10 mg/kg) or PBS (control) starting 2 h before intravenous inoculation of *S. aureus* Newman strain (2 × 10^6^ CFU/mouse). Treatment with peptides or PBS was then continued every day, twice/day, until animals were euthanized on day 7. (**A**) Weight development over the seven-day monitoring. (**B**) Bacterial load in kidneys (CFU) on day 7 post-infection (n = 10 (4 died)). (**C**) Mortality of mice infected with *S. aureus* Newman. P-values determined using Mann–Whitney U-test with data expressed as the mean ± standard error of the mean (A) or median (B). * *P* < 0.05, ** *P* < 0.01.

The mice infected with *S. aureus* Newman began to lose weight after infection. In the L15-treated mice, weight loss was significantly reduced in L15 treated compared to control mice (Fig. 6A). No significant difference was observed with regard to the bacterial load in the kidneys (Fig. 6B). Regarding the mortality rate, 40% of the animals in the control group died, whereas all mice treated with L15 survived during the 7-day observation period (Fig. 6C), indicating that L15 treatment significantly decreased lethality of *S. aureus* bacteremia.

### L15 and L13 influence the response of innate immune cells to *S. aureus*

To understand the molecular basis of the protective effect of L15 and L13 we carried out immune stimulation studies with human peripheral blood mononuclear cells (PBMCs) from buffy coat blood samples of healthy donors. We tested the ability of the two peptides to induce IL-6, IL10, IL-12p70, IL12-p40, IL-23, TNFα, IL-1ß, IL-1RA, IP-10, and TARC. L15 was relatively inert with respect to cytokine inducing activity in PBMCs (Fig. S3).

**Figure 7.**
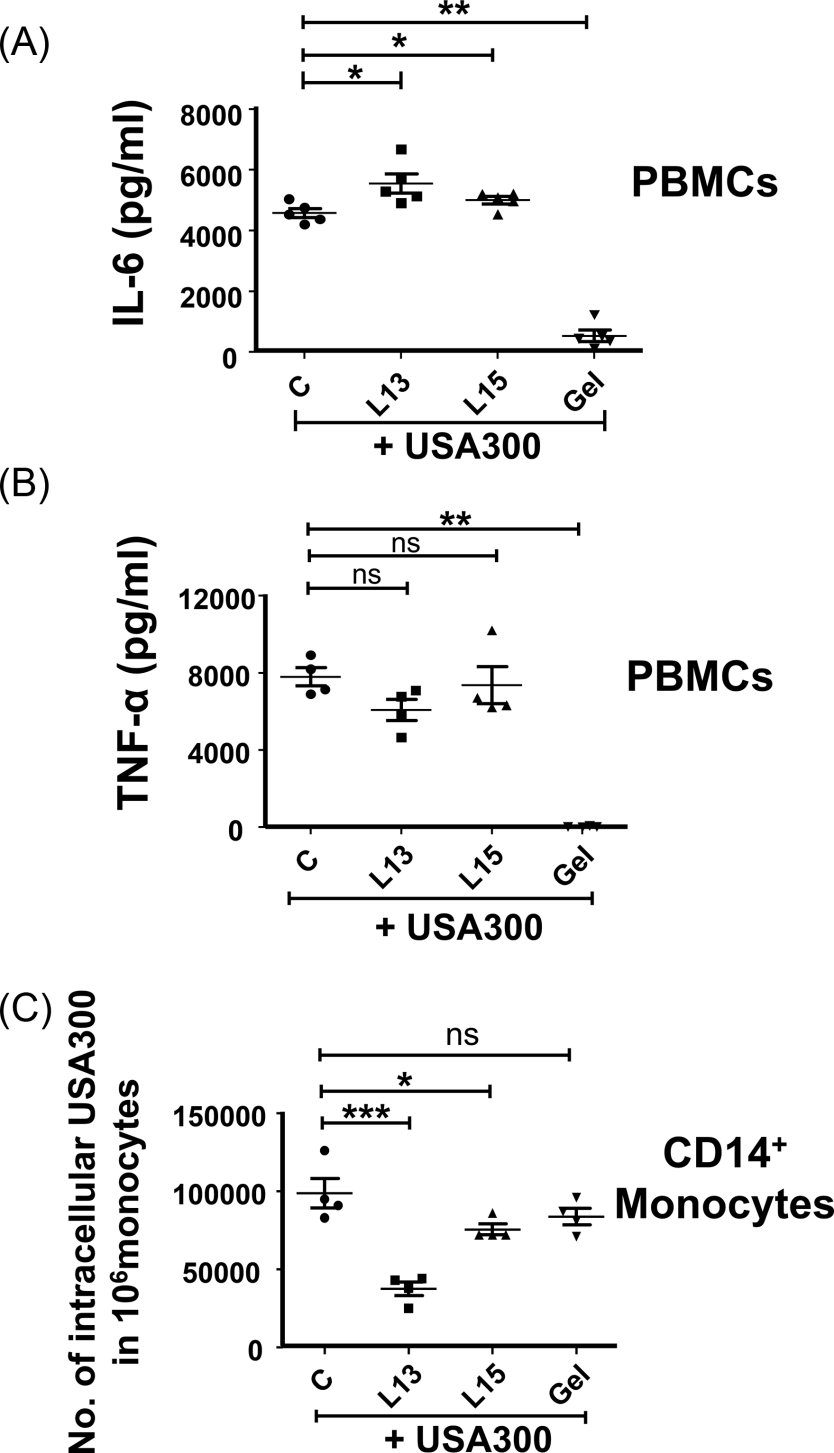
Influence of L13 and L15 on the response of host innate immune cells. Release of IL-6 (**A**) and TNF-alpha (**B**) in the supernatant of *S. aureus*-infected PBMCs was assayed by ELISA 20 h after stimulation with L13 or L15 or geldanamycin. C indicates control cells without peptide pretreatment. Samples from four donors were carried out in triplicate. (**C**) Effect of L15 and L13 on *S. aureus* USA300 phagocytosis by primary human CD14^+^ monocytes; control (C) was without peptide pretreatment. Samples from 4 donors were carried out in duplicate. Error bars represent SEM. Statistic significances were calculated between the peptide treated cells compared to control (C) by using one-way ANOVA analysis with Tukey's multiple comparison test: *P < 0.05, **P < 0.01, ***P < 0.001, ns > 0.05.

We then stimulated *S. aureus* USA300 infected PBMCs from four to five healthy donors with L15 or L13 (20 and 30 µM, respectively) and compared it to the unstimulated USA300 infected PBMCs. It turned out that both peptides significantly increased IL-6 (n = 5) but not TNF-alpha production (n = 4) (Fig. 7A and B). Geldanamycin (5 µM) inhibited both the production of IL-6 and TNF-alpha in infected PBMCs. Later, we examined whether the peptides affected phagocytosis of *S. aureus*. The phagocytosis was performed in CD14^+^ monocytes purified from PBMCs (n = 4). The assay revealed that both peptides significantly decreased phagocytosis of *S.aureus* into CD14^+^ monocytes: L15 by about 22.6 ± 7.67%geldanamycin and its derivatives and L13 by about 62.0 ± 6.80% (Fig. 7C). Geldanamycin (5 µM) showed no significant impact on phagocytosis.

### Multiple sequence alignment identifies Lpl1 homologs in other species

**Figure 8.**
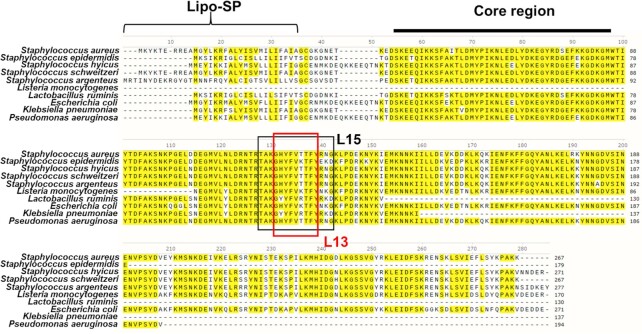
Multiple sequence alignments of Lpl1 from *S. aureus* USA300 with other bacteria. These include *Staphylococcus epidermidis* SE62, *Staphylococcus hyicus* NCTC 8294, *Staphylococcus schweitzeri* NCTC 13712, *Staphylococcus argenteus* B3-25B, *Listeria monocytogenes* ATCC 15313, *Ligilactobacillus ruminis* ATCC 27780, *Escherichia coli* NCTC 9001, *Klebsiella pneumoniae* NCTC 9633 and *Pseudomonas aeruginosa* PA216. The lipoprotein signal peptide is indicated by the bracket, the conserved core region by the bar and the L15,L13 sequences are boxed.

We also investigated in which microorganisms Lpl1 homologs occur. Protein BLAST analysis revealed that Lpl1 proteins with high similarity are present not only in different staphylococcal species but also in different unrelated bacterial species (Fig. 8). Lpl1 homologs from *Staphylococcus hyicus, Staphylococcus schweitzeri*, and *Staphylococcus argenteus* showed identities of 88%, 97%, and 88%, respectively, and the corresponding L15 and L13 homologous sequences in these lipoproteins were identical. The Lpl1-homolog was also found in some *Staphylococcus epidermidis* strains, however, the corresponding L15 sequence was not exactly the same. *Listeria monocytogenes* had an Lpl1 homolog with a sequence similarity of 80% and *Ligilactobacillus ruminis* with 74%. Even Gram-negative species such as *Escherichia coli, Klebsiella pneumoniae*, and *Pseudomonas aeruginosa* had a very similar lipoprotein with % sequence similarity of 75, 90, and 85, respectively. Interestingly, the corresponding L15 sequence of *P. aeruginosa* was identical to that of *S. aureus*.

Lpl1 protein was blasted against all organisms. We obtained similarities only against bacteria and within the bacteria only against these pathogenic species. The limitation to these species suggests that Lpl protein plays an important role in the pathogenicity and/or fitness of these bacteria, which should be further investigated.

## Discussion

Hsp90 is a molecular chaperone that ensures cellular proteostasis by folding, stabilizing, activating and degrading over 400 client proteins (Hoter et al. 2018). There exist two isoforms, Hsp90α and β, however, because of the structural and functional similarity the name HSP90 is normally used for both (Hoter et al. 2018). Normally, Hsp90α localizes in the cytoplasm but it can be secreted under stress such as reactive oxygen species, heat, hypoxia, irradiation, or tissue injury (Jackson 2013). The extracellular form, eHsp90α, has been shown to enhance cell motility and support wound healing (Li et al. 2012). Due to its ability to affect numerous client proteins, inhibition of Hsp90 is regarded as an attractive approach for cancer treatment (Sanchez et al. 2020). Therefore, any new compound that interacts with and alters the activity of Hsp90 is regarded with great interest.

The well-studied geldanamycin (Blagosklonny 2002, Miyata 2005) and our L15 and L13 peptides have one thing in common, namely binding to Hsp90. This was the reason why we always included geldanamycin as a control in our studies. However, beyond the binding to Hsp90 the similarities become less pronounced. For example, Lpl1 increased both the internalisation of *S. aureus* by host cells and F-actin levels, whereas geldanamycin decreased the internalisation and F-actin levels by about the same factor suggesting that they bind to different sites of Hsp90 (Tribelli et al. 2020).

In the search for Lpl1 domains that interacted with Hsp90α, we found 2 peptides, L15 and L13, which interacted with Hsp90α but, unlike the parent protein Lpl1, decreased the internalisation of *S. aureus* into host cells, similar to geldanamycin does. L15 and L13 are part of a ß-sheet domain in Lpl1 as illustrated in Fig. 1B and C. The previously described L27 peptide (38 aa long) is localized in the C-terminal alpha-helical and loop structures part of Lpl1 and activates, like the parent protein Lpl1, the internalisation of *S. aureus*. Hsp90 comprises three main conserved domains, the N-terminal domain (NTD), C-terminal domain (CTD), and middle domain (MD) each performing a specific function (Hoter et al. 2018). While geldanamycin is known to bind to the N-terminal NTD domain thereby inhibiting the ATPase activity that is necessary to regulate Hsp90 conformation (Grenert et al. 1997, Gorska et al. 2012), we currently do not know how or where L13/L15 interact with Hsp90.

Owing to the significant roles of protein kinases and phosphatases in cellular regulation (Cheng et al. 2011) we also investigated whether L15 or L13 exerted an effect on total ATPase activity in HaCaT cells. However, we could not detect any significant inhibition by L15 or L13 and assumed that Hsp90 ATPase activity is not significantly affected (Fig. S4). Most likely the peptides bind to a different site on Hsp90 than geldanamycin.

Similar to geldanamycin, L15 and L13 inhibit F-actin formation and *S. aureus* internalisation in host cells (Fig. 2 and Table 1).

Since geldanamycin is more cytotoxic than L15 and L13 we took care of using it at the subinhibitory concentration of 5 µM, while L15 and L13 were used at concentrations of ≈ 20 and 30 µM, respectively. In both the *G. mellonella* and the mouse model, L15 reduced the lethality of *S. aureus*, by about 30% in the insect model and about 40% in the mouse model (Figs 5 and 6). In the insect model the peptides were added immediately before infection with *S. aureus*. In the mouse model, L15 was added 1 h before the infection with *S. aureus* and at daily time intervals, similar to classical antibiotic treatment. This positive effect, shows that the peptides act not only at the cellular level but also in animal models.

In order to reduce the lethality of *S. aureus*, we speculated that (i) L15 and L13 inhibited growth and expression of virulence factors of *S. aureus* or (ii) they strengthened the host defense or (iii) both.

At the concentrations used, L15 and L13 neither inhibit the viability of host cells (Fig. S1A and B) nor the growth or hemolytic activity of *S. aureus* (Fig. S2A and B). But, it was observed that the peptides L15 and L13, can interact directly not only to Hsp90α, but also to the Lpl1 (Fig. 3A and B).

We also believe that they strengthen host defenses through their interaction with Hsp90. However, there might be also a third mechanism. In the blood stream there are abundant neutrophils who can sense, engulf, and kill the bacteria. The inhibitory effect of L15 on bacterial internalization in keratinocytes and monocytes may also apply to the endothelial cells. In the bacteremia model, less internalized bacteria upon L15 treatment may result in higher number of bacteria in the blood stream that are more susceptible to immune killing which may lead to less focal infection in the vital organs.

How the defense is strengthened is not completely clear. However, insects and mammalians have one defense system in common, namely the innate immune system (Sheehan et al. 2018). Both peptides are relatively inert with respect to cytokine inducing activity in PBMCs (Fig. S3). However, *S. aureus* infected PBMCs that were pretreated with L13 and L15 showed an increase in IL-6 production (Fig. 7A), and in primary human monocytes pretreatment with L13 or L15 decreased the *S. aureus* internalization (Fig. 7B). In the latter case, the peptides could compete with the Lpl proteins on the surface of *S. aureus* for binding to the Hsp90 receptor or bind directly to the Lpl preventing them from interacting with Hsp90 (Fig. 3A and B). Both could lead to neutralization of the Lpl proteins, and this in turn could lead to reduced pathogenicity. This would be consistent with our previous results showing that deletion of the *lpl* genes significantly reduces the pathogenicity of *S. aureus* in a mouse kidney abscess model (Nguyen et al. 2015).

Hsp90 and related heat shock proteins are also involved in host defence (Calderwood et al. 2016). Hsp90 induces the adaptive immunity by activation of antigen presenting cells and dendritic cells. And the related heat shock protein, GRP94 (gp96), shows the same domain structure as Hsp90 and also binds geldanamycin. It is the most abundant glycoprotein in the ER hence known as endoplasmin. The remarkable feature of GRP94 is that some of its client proteins are important components of the immune system such as TLR1, 2, 4, and MHC class II (Schaiff et al. 1992, Randow and Seed 2001, Staron et al. 2010, Mesquita et al. 2017). In fact, GRP94 (GP96) is the master chaperone for Toll-like receptors and is important in the innate function of macrophages (Yang et al. 2007).

## Conclusion

Any compound that interacts with Hsp90 and thereby exerts an effect on cellular physiology is of particular interest. The Lpl1-derived small peptides, L15 and L13, not only impact the cytoskeleton and the associated internalization of *S. aureus* by the host cells *in vitro*. They also exert an effect in both insect and mammalian models by reducing the lethality of *S. aureus* infection. How this happens is still unclear, however, we envisage two scenarios: (i) binding of the peptides to Hsp90 and related Hsp proteins engages the innate immune system in such a way that it responds faster or more strongly to *S. aureus* infection, or (ii) binding of the peptides to Lpl1 interferes the binding of the membrane-anchored Lpl proteins of *S. aureus*. Indeed, we found that the peptides also interact with Lpl1. This indicates that the peptides bind to both Lpl1 and Hsp90. We now hypothesize that the L15/L13-induced reduction in virulence of *S. aureus* is due to interaction with both the Lpl protein and Hsp90. Whether both interactions or only one of them affects virulence will have to be further investigated. In this work, we also demonstrate that rationally selected peptides of a cell surface-bound virulence factor can turn out to be promising drugs.

## Supplementary Material

uqac023_Supplemental_Files
