# Supplementary figures and images for: From an Hsp90 - binding protein to a peptide drug
Source: Microlife. 2022 Dec 13;4:uqac023. doi: 10.1093/femsml/uqac023 (PMC10117725; doi:10.1093/femsml/uqac023)

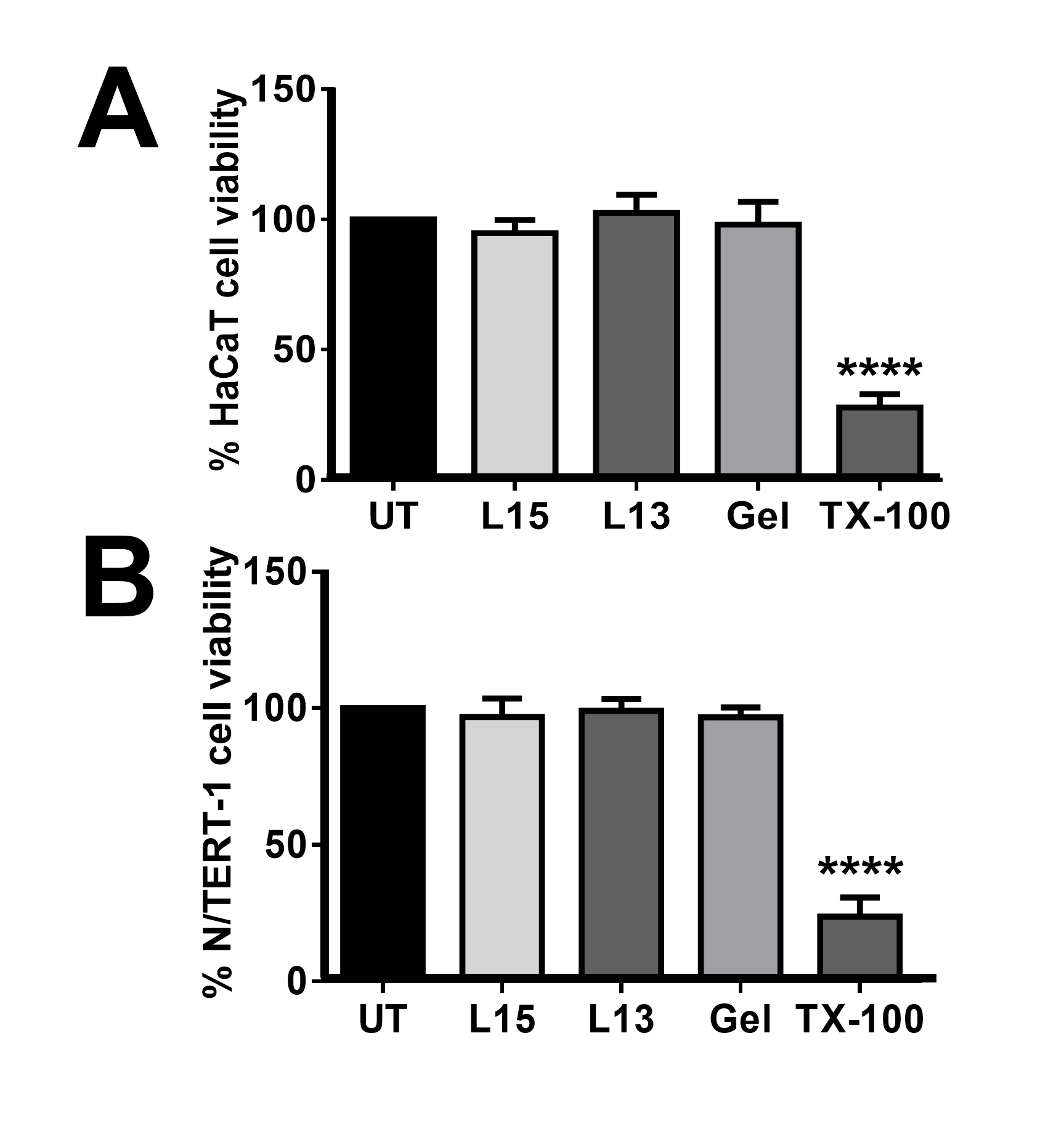

Supplement: uqac023_Supplemental_Files [file uqac023_supplemental_files.zip › Fig. S1_Supplementary Data.tif]

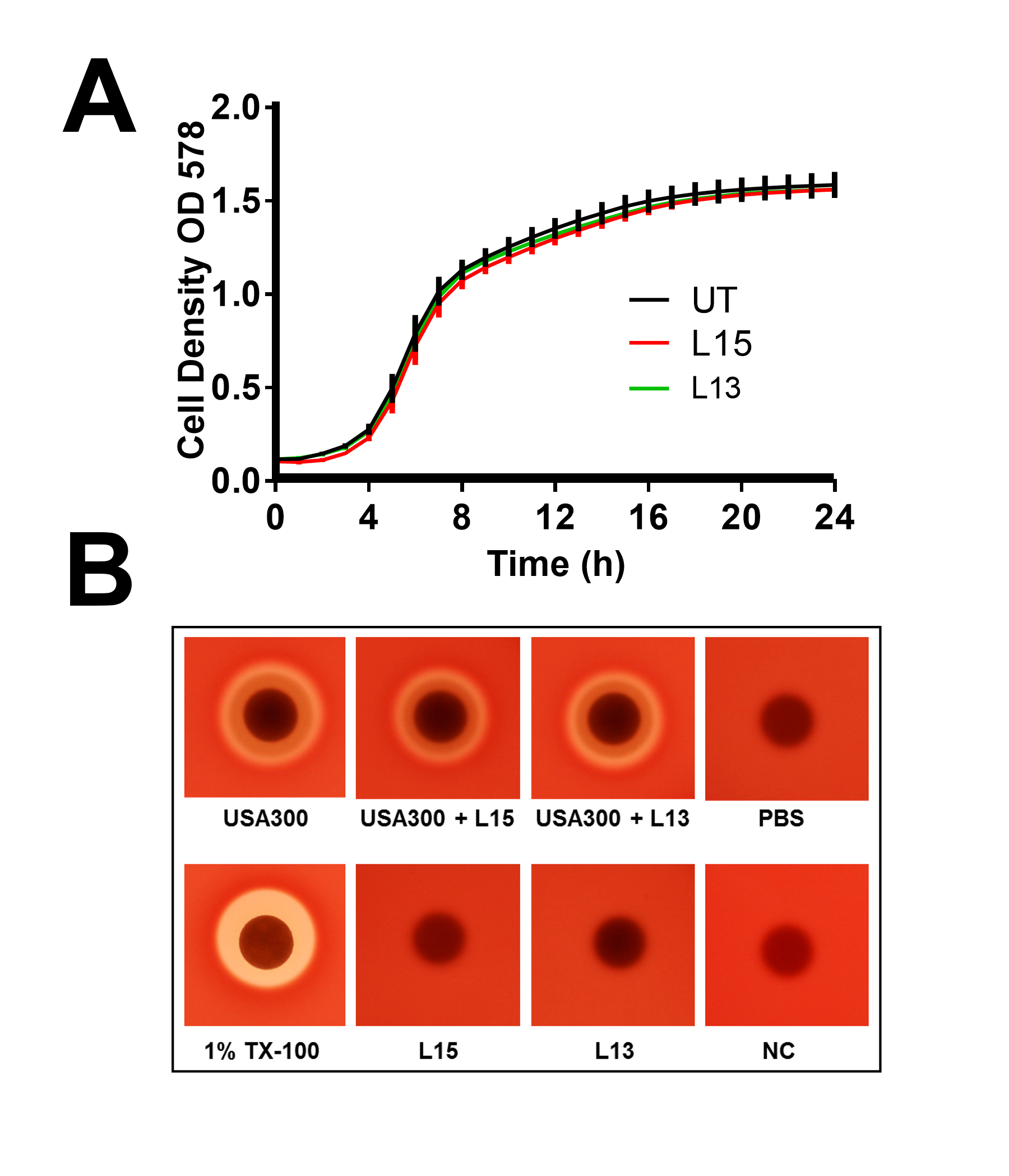

Supplement: uqac023_Supplemental_Files [file uqac023_supplemental_files.zip › Fig. S2_Supplementary Data.tif]

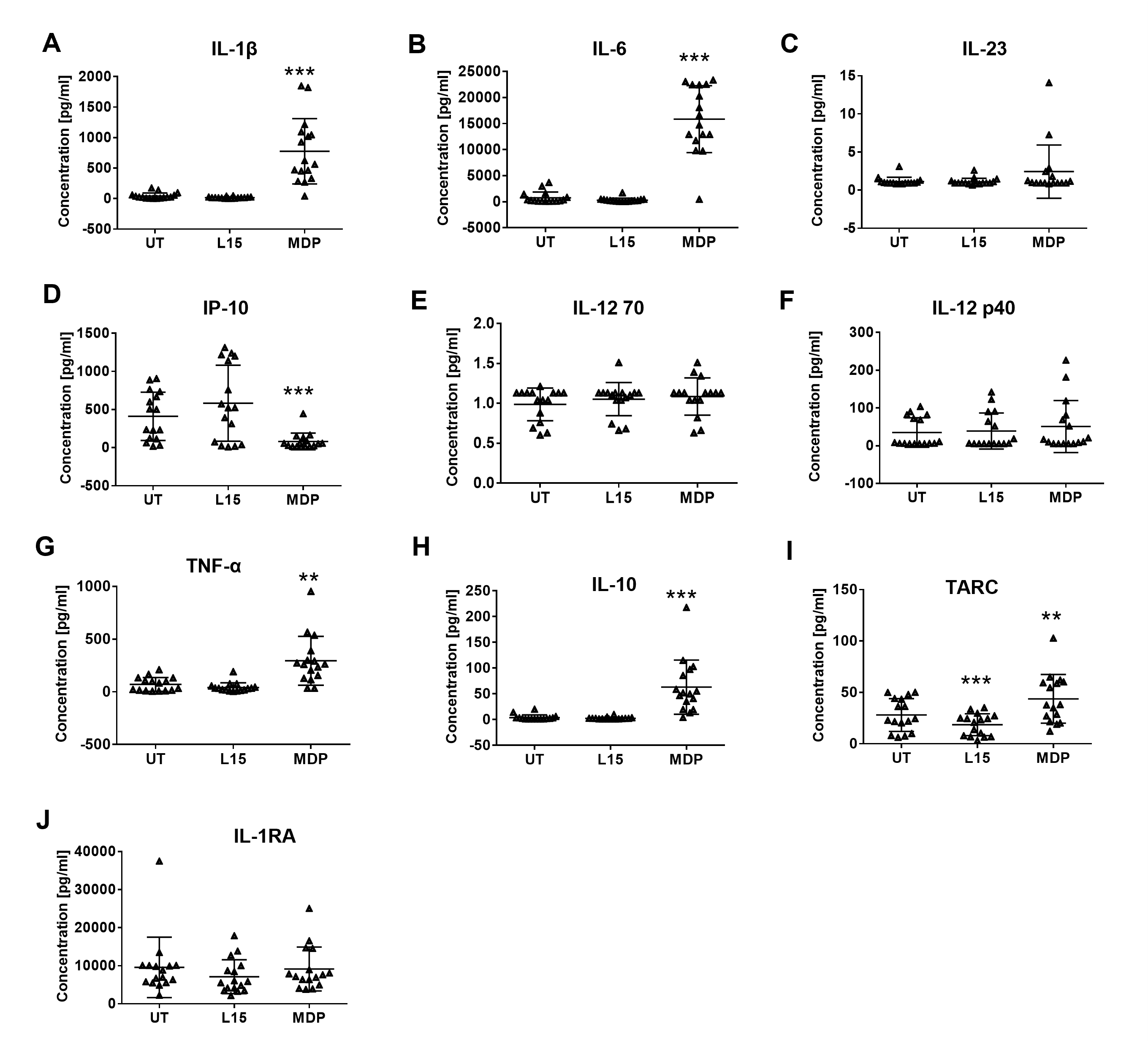

Supplement: uqac023_Supplemental_Files [file uqac023_supplemental_files.zip › Fig. S3_Supplementary Data.tif]

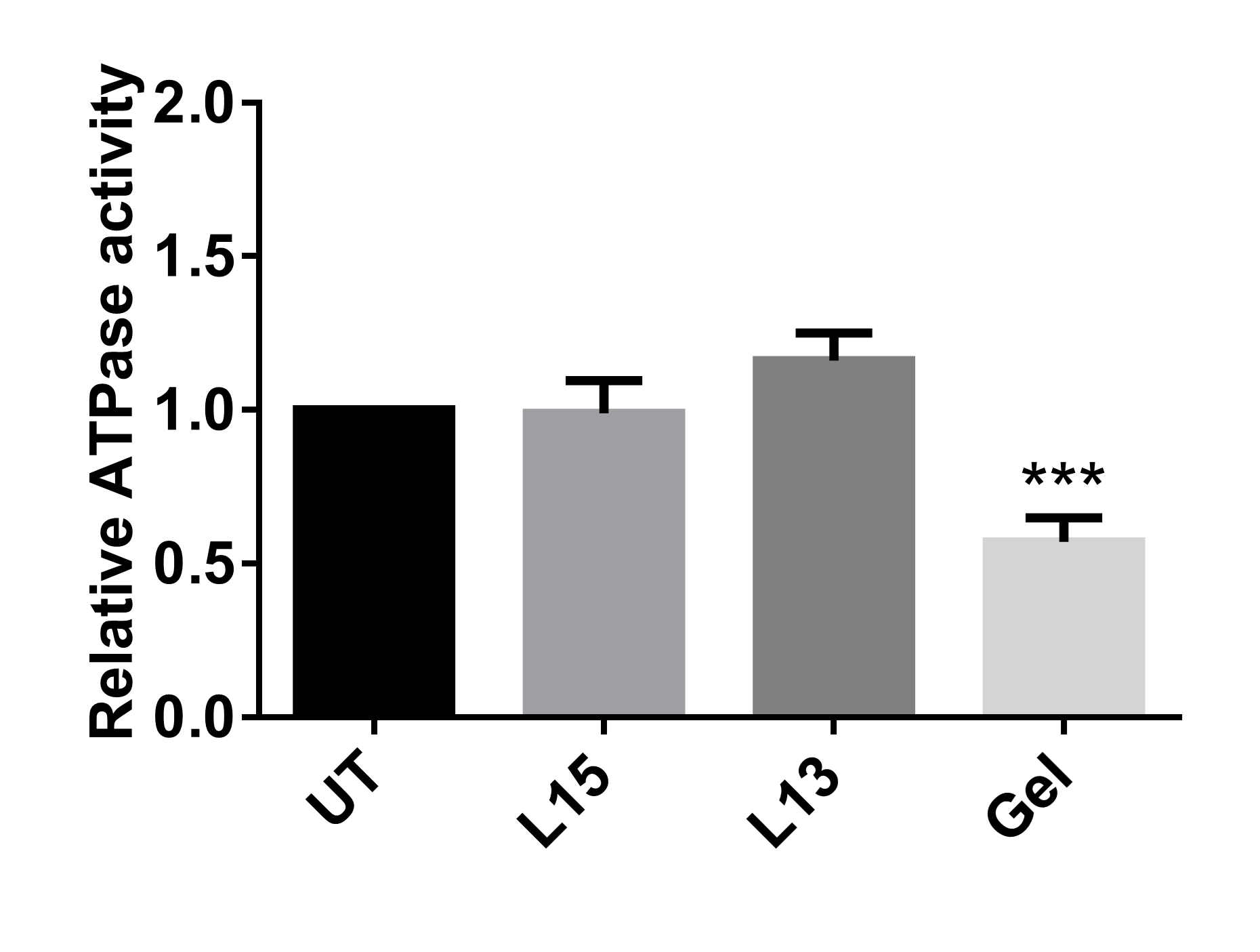

Supplement: uqac023_Supplemental_Files [file uqac023_supplemental_files.zip › Fig. S4_Supplementary Data.tif]
